# Development and Psychometric Validation of the Health Professionals’ Job Satisfaction Scale

**DOI:** 10.3390/healthcare13222917

**Published:** 2025-11-14

**Authors:** Ana Lúcia João, Paula Chaves, Ana Prata Massano, Fátima Diogo, Rita Paulos, António Portelada, João Alves

**Affiliations:** 1 School of Health, Santarém Polytechnic University, 2001-904 Santarém, Portugal; 2 Comprehensive Health Research Center, Universidade de Évora, Palácio dos Colegiais 2, 7004-516 Évora, Portugal; 3 Lezíria Local Health Unit, 2005-177 Santarém, Portugaljoao.alves@ulsleziria.min-saude.pt (J.A.); 4 School of Education, Santarém Polytechnic University, 2001-904 Santarém, Portugal; antonio.portelada@ese.ipsantarem.pt

**Keywords:** healthcare professionals, scale validation, organisational quality, psychometric assessment

## Abstract

**Background/Objective**: Job satisfaction is a key determinant of healthcare professionals’ well-being, quality of care, and organisational performance. In Portugal, although validated tools exist for nurses, there is no comprehensive instrument for different professional groups. This study aimed to develop and validate the Health Professionals’ Job Satisfaction Scale (ESPST—Escala de Satisfação dos Profissionais de Saúde no Trabalho), designed to assess job satisfaction across diverse healthcare categories. **Methods**: A quantitative, descriptive, cross-sectional study was conducted with 549 professionals from a Portuguese hospital. The ESPST was developed from literature review and expert focus groups, comprising 50 Likert-scale items. Construct validity was examined using exploratory factor analysis (EFA), and reliability was assessed via Cronbach’s alpha. **Results**: The EFA revealed an eight-factor structure, explaining 70.3% of total variance. The KMO value was 0.963, and Bartlett’s test of sphericity was significant (*p* < 0.001). The factors were: leadership and management, nature of work, colleagues, recognition by service users, career progression, human resources, institutional protocols, and peer recognition. Cronbach’s alpha was 0.973 for the total scale, with subscales above 0.84, indicating excellent reliability. **Conclusions**: The ESPST is a valid and reliable instrument for assessing job satisfaction among healthcare professionals across different categories. Its multidimensional scope allows clinical and research applications, supporting organisational strategies to improve professional well-being and quality of care. Future studies should include confirmatory analyses to strengthen its psychometric robustness.

## 1. Introduction

Job satisfaction represents an attitude, emotion, or feeling arising from a worker’s evaluation of various dimensions of their work that are personally meaningful, taking into account their expectations, achievements, and investments within the work context. It encompasses interpersonal relationships and comparisons with others and results from the attainment of outcomes or rewards. Job satisfaction directly influences health, quality of life, and work-related behaviours, with effects extending to other colleagues and the institution [1]. In healthcare organisations, this construct is a fundamental pillar for institutional efficiency, overall performance, and the quality of care provided to service users [2]. The degree of satisfaction experienced by professionals reflects not only workplace conditions but also the recognition and appreciation of their contributions within the healthcare system [2,3], exerting a significant influence on productivity, motivation, and team well-being [3].

Active involvement of professionals in institutional dynamics and acknowledgement of their contributions foster higher levels of job satisfaction, which in turn translate into improvements in care quality and organisational culture [4,5]. Several factors have been identified as determinants of satisfaction in healthcare settings, including working conditions, interpersonal relationships, workload, team dynamics, leadership style, remuneration, professional development, and opportunities for recognition and participation in decision-making [1,6,7]. Thus, the assessment of job satisfaction emerges as an indispensable component for monitoring and continuously improving quality standards within healthcare institutions [8].

Job satisfaction is a multidimensional and cross-cutting phenomenon that encompasses all professional categories in healthcare. It correlates strongly with individual and collective performance, influencing motivation, organisational commitment, and the effectiveness of multidisciplinary teams [3,9]. Nonetheless, it remains a complex reality, influenced by subjective factors such as socio-professional context, mental health, self-esteem, and perceptions of physical and emotional well-being [10]. In Portugal, research has predominantly focused on nurses [11,12,13,14], whose role is central to patient care and institutional functioning [15,16]. Although some studies have addressed other professional groups, the literature reveals a shortage of integrated research encompassing the multiple professional categories that compose the healthcare sector [17].

Internationally, several instruments have been developed to assess job satisfaction in general occupational contexts, such as the Job Satisfaction Survey and the Minnesota Satisfaction Questionnaire. However, systematic reviews [18] have shown that these instruments are conceptually limited when applied to healthcare settings, as they often overlook critical aspects such as interprofessional collaboration, emotional demands, patient recognition, and adherence to institutional protocols—dimensions intrinsic to healthcare work [1,4]. In Portugal, the only validated instrument available to date is the Nurses’ Job Satisfaction Scale (ESET), developed by João et al. (2017) [14], which restricts its applicability to nursing professionals. This highlights the need for a validated, multidimensional tool capable of assessing job satisfaction across the diverse professional categories that comprise the healthcare sector.

Given this gap, the present study aimed to develop and validate the Health Professionals’ Job Satisfaction Scale (ESPST—*Escala de Satisfação dos Profissionais de Saúde no Trabalho*). The ESPST was designed to assess job satisfaction across different professional groups, integrating the specificities of healthcare practice and offering a comprehensive, psychometrically sound instrument for research and organisational use. Its development was based on an extensive literature review, critical analysis of existing tools (including the ESET) [15], and consultation with a focus group of healthcare experts. By providing a reliable and valid instrument, this study contributes to promoting professional well-being, improving organisational quality, and supporting evidence-based management strategies within healthcare institutions.

## 2. Materials and Methods

### 2.1. Study Design

This study adopted a quantitative, descriptive, cross-sectional design, with the primary aim of constructing and psychometrically validating the Healthcare Professionals’ Job Satisfaction Scale (ESPST). The research was conducted within the positivist paradigm, focusing on the objective measurement of variables and statistical analysis of questionnaire-based data.

**Figure 1:**
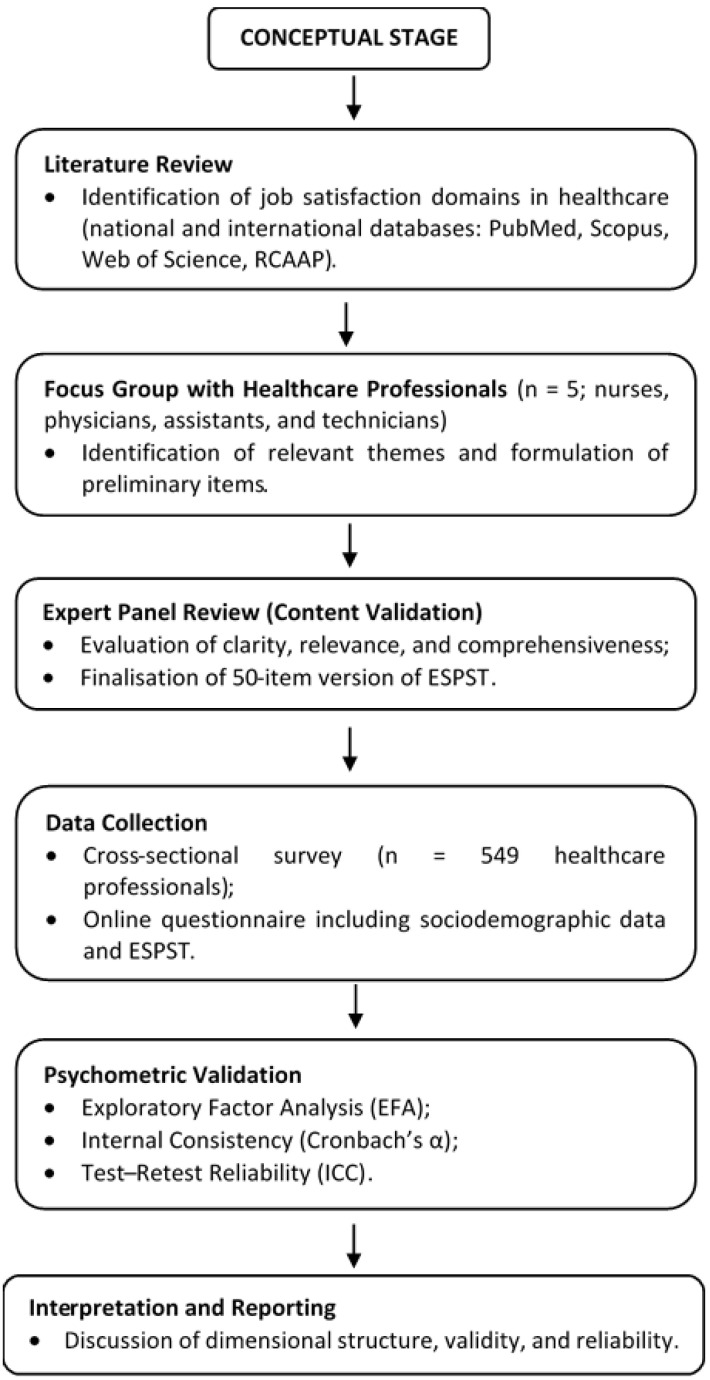
Flowchart of the methodological stages of the study.

To ensure methodological transparency and reproducibility, the entire research process was structured into five sequential stages, as illustrated in Figure 1. These stages encompassed the conceptual development of the instrument, data collection, and the psychometric validation procedures.

### 2.2. Population and Sample

The study population consisted of all healthcare professionals working in a public hospital integrated into the Lezíria Local Health Unit, located in central Portugal, totalling 1732 individuals at the time of data collection.

A non-probabilistic, convenience sampling approach was adopted, including all professionals who met the following inclusion criteria: (i) employment relationship with the institution during the data collection period; (ii) performance of clinical, technical, or administrative functions related to healthcare delivery; and (iii) voluntary agreement to participate in the study. Exclusion criteria included being on long-term leave (medical or maternity).

Participants were invited via institutional e-mail, containing a brief explanation of the study objectives, ethical assurances, and a link to the online questionnaire (Google Forms). Two reminders were sent at one-week intervals to encourage participation. Data collection occurred over a two-month period, between 18 April and 18 June 2023.

A total of 549 valid responses were obtained, corresponding to a 31.69% response rate, which ensured heterogeneity across professional categories and departments. This approach, although non-probabilistic, provided adequate internal variability to allow psychometric analyses and meaningful inferences within the organisational context.

This transparent and replicable recruitment strategy enhances the reliability of the study design and allows similar investigations to be conducted in comparable healthcare institutions.

### 2.3. Instrument Development

The development of the Healthcare Professionals’ Job Satisfaction Scale (ESPST) followed a structured methodological process comprising three stages: (i) a literature review, (ii) focus group discussions, and (iii) expert content validation.

First, a comprehensive national and international literature review was conducted to identify the main domains and determinants of job satisfaction in healthcare contexts. This review provided the theoretical foundation for the initial pool of items. Between January and March 2023, searches were performed in the databases PubMed, Scopus, Web of Science, and RCAAP, using the keywords *“job satisfaction”*, *“healthcare professionals”*, *“scale”*, and *“validation”*. Articles published between 2010 and 2023, in Portuguese or English, were included if they described instruments assessing job satisfaction in healthcare settings. Studies that did not address psychometric evaluation or were unrelated to healthcare professions were excluded.

In the second stage, a focus group was conducted with five healthcare professionals representing different categories—including nurses, physicians, operational assistants, and diagnostic and therapeutic technicians—selected by convenience to ensure diversity of professional perspectives and hierarchical levels. The session, lasting approximately 60 min, was moderated by a researcher experienced in qualitative methods. Participants discussed factors influencing their satisfaction at work. The themes that emerged informed the formulation of 50 preliminary items, expressed as statements through which professionals could indicate their level of satisfaction with various aspects of their work environment.

Finally, the preliminary items were reviewed by a panel of experts for content validation, assessing relevance, clarity, and comprehensiveness. The final version of the ESPST encompassed dimensions such as interpersonal relationships, leadership, recognition, nature of tasks, working conditions, work–life balance, organisational management, and human resources. This structure was subsequently tested through exploratory factor analysis to evaluate the instrument’s construct validity.

### 2.4. Procedure and Data Collection Instruments

Data for this cross-sectional study were collected between April and June 2023 from a sample of healthcare professionals working in a Portuguese hospital unit. Data collection was conducted using a structured online questionnaire (Google Forms), carefully designed to capture both sociodemographic/professional characteristics and job satisfaction.

The questionnaire consisted of two sections:

- Sociodemographic and professional variables—including age, gender, marital status, number of children, academic qualifications, length of service, profession, type of contract, department, work schedule, among others. These were selected based on previous literature highlighting the influence of sociodemographic and professional factors on job satisfaction among healthcare workers.
- Healthcare Professionals’ Job Satisfaction Scale (ESPST)—developed from an extensive literature review and adapted to the Portuguese hospital context. The ESPST comprises 50 items, scored on a five-point Likert scale (“not at all”, “a little”, “moderately”, “very”, “extremely”) to evaluate satisfaction across different workplace dimensions (e.g., nature of work, interpersonal relations, working conditions, professional recognition). To enhance validity, an additional “not applicable” option was introduced following the pre-test, enabling respondents to omit items irrelevant to their role.

### 2.5. Ethical Considerations

Ethical procedures and authorisation to conduct the study were obtained from the Hospital Administration Board and the Institutional Ethics Committee (Opinion No. 22/2023). Data collection ensured participant confidentiality and anonymity, with respondents being informed that their data would be used exclusively for research purposes. They were also informed that participation carried neither costs nor financial benefits and that they could withdraw from completing the questionnaire at any stage, as participation was entirely voluntary.

### 2.6. Statistical Analysis

Data analysis was performed using IBM SPSS Statistics v29, adopting a significance level of 5% (*p* ≤ 0.05).

The psychometric validation of the ESPST involved:

- Exploratory Factor Analysis (EFA): to identify the underlying factor structure. Principal Component Analysis (PCA) with orthogonal Varimax rotation was applied. Sampling adequacy was assessed using the Kaiser-Meyer-Olkin (KMO) index, and Bartlett’s test of sphericity was performed. Factor retention followed Kaiser’s criterion (eigenvalues > 1). Items with factor loadings below 0.30 were excluded.
- Construct validity: assessed via Spearman’s correlation coefficients between extracted factors, examining the consistency of the multidimensional structure.
- Internal consistency: Cronbach’s alpha coefficients were calculated for the overall scale and each subscale. Item–total correlations and alpha if item deleted were also examined.
- Test–retest reliability: Stability was assessed using the intraclass correlation coefficient (ICC), considering both single and average measures, with 95% confidence intervals and associated F-tests.

## 3. Results

### 3.1. Sociodemographic and Professional Description of the Sample

A total of 549 healthcare professionals participated in the study, representing 31.69% of the hospital’s workforce. The majority were female (82.9%), aged between 30 and 49 years (57%), and married or in a civil partnership (63.2%). Most participants held a bachelor’s degree (58.8%), had stable employment contracts (62.1% permanent), and an average professional experience of 6–10 years (16.0%) (Table 1).

Regarding professional roles, nurses constituted the largest group (50.8%), followed by operational assistants (19.3%) and physicians (15.7%). The most common departments were internal medicine (29.9%), support services (16.0%), surgery (13.5%), and emergency (13.3%). The majority provided direct patient care (85.6%), worked 35–40 h per week (91.1%), and lived within 30 km of the hospital (80.3%).

These findings confirm the internal diversity of the sample, ensuring representativeness across professional categories and organisational areas, while reflecting the demographic profile typical of Portuguese hospital institutions (Table 1).

### 3.2. Exploratory Factor Analysis and Dimensional Structure of the ESPST

The psychometric characteristics of the ESPST were examined in the sample of 549 participants. The suitability of the data for factor analysis was verified using the Kaiser–Meyer–Olkin (KMO) measure of sampling adequacy and Bartlett’s test of sphericity. The KMO value obtained was 0.963, which is considered excellent. Bartlett’s test produced a χ^2^ (1225) = 23,421.495, statistically significant at *p* < 0.001.

Factor analysis is a statistical procedure that reduces the complexity of the original problem by grouping variables (in this study, items 1–50 of the ESPST) into clusters formed by strongly correlated variables. These groups originate distinct factors or latent variables, which are random variables, ideally fewer in number than the original set of observed variables [19].

An exploratory factor analysis of the ESPST (Table 2) yielded eight factors with eigenvalues greater than 1, according to Kaiser’s criterion [19,20]. Together, the eight retained factors explained 70.31% of the total variance.

The first factor accounted for 49.49% of the total variance, with an eigenvalue of 21.760, and was composed of 14 items. The second factor explained 6.75% of the total variance, with an eigenvalue of 3.74, comprising 7 items. The third factor, with an eigenvalue of 2.71, accounted for 5.42% of the variance and contained 6 items. The fourth factor, with an eigenvalue of 1.95, explained 3.98% of the variance and included 4 items. The fifth and sixth factors, both composed of 6 items, explained 3.53% and 2.96% of the variance, respectively. The seventh factor, comprising 4 items with an eigenvalue of 1.11, explained 2.21% of the variance. The final factor, with 3 items, explained 2.03% of the variance and had an eigenvalue of 1.01 (Table 2).

The principal component analysis with Varimax rotation yielded the factor loadings shown in Table 3.

Following the exploratory factor analysis, the factors were presented as dimensions of the ESPST. It was observed that some items had high communalities, such as item 16 (h^2^ = 0.862), item 15 (h^2^ = 0.864), and item 31 (h^2^ = 0.836). These values indicate that these items are well represented by the extracted factors, with a high degree of variability explained by the factorial structure. The strong association of these items with the extracted factors reinforces their relevance and reliability within the measure in question, suggesting that they are fundamental for representing the underlying dimensions the scale aims to measure (Table 3).

On the other hand, some items showed intermediate communalities, such as item 9 (h^2^ = 0.741) and item 5 (h^2^ = 0.724). While these items have a substantial portion of their variability explained by the factors, a significant portion of this variability is not captured by the extracted factors. This suggests that these items may be influenced by additional factors or characteristics not directly addressed by the factor analysis, which could indicate the need for a revision of the model or the inclusion of new factors for a more comprehensive representation.

Items with lower communalities, such as item 43 (h^2^ = 0.40), show that the variability of these items is only partially explained by the extracted factors. These values suggest that these items may exhibit more independent behaviour.

The first dimension, designated as satisfaction with leadership and management, consisted of 14 items. The name attributed to this factor took into consideration the fact that most of its items assess professionals’ satisfaction with management and supervisors, emphasising their influence on organisational culture, on motivation and professional development of the team, and on the full involvement of staff in the objectives of the organisation [13,21,22]. Each of the evaluation items contributes to a holistic understanding of the relationship between managers and other staff members, providing valuable information for strategic decision-making, in line with other authors who highlight the influence of the relationship between subordinates and managers on job satisfaction [23].

The second factor was designated satisfaction with the nature of work, as the items that comprise it enable the evaluation of satisfaction related to the professional activity performed (Table 4), in accordance with the findings of other authors [11,12,13,21]. The common focus of health professions is the individual in need of healthcare, and thus the nature of healthcare professionals’ work is the provision of care, within the responsibilities and competences specific to each profession [24,25,26].

The third factor, satisfaction with colleagues, consisted of 6 items referring to the interpersonal relations established with colleagues (Table 4), also identified in the literature [21,24,27]. Interpersonal relationships in the workplace are presented in the literature as determinants of burnout syndrome [28]; therefore, when experienced as satisfactory, they act as protective factors against it. Positive interpersonal relations are associated with job satisfaction [29].

The fourth factor of the ESPST was designated satisfaction with service users’ recognition, in which all 4 items are related to the perception of being valued by service users for the functions performed (Table 4). The literature includes studies on service users’ satisfaction with the work carried out by healthcare professionals [1,21], which attribute value to it. Transposed into practice contexts, this justifies the inclusion of these items within this dimension.

The fifth factor, composed of 6 items, was called satisfaction with functions and career progression, as the items refer to satisfaction with the functions exercised and with the opportunity for career advancement (Table 4). Other authors [12,21,27] also address aspects of this dimension, usually reporting low to moderate levels of satisfaction.

The sixth factor, satisfaction with human resources, was composed of 6 items that assess participants’ perceptions of the number of professionals available, considering the demands and the number of service users requiring care (Table 4). This aspect is also noted in other studies [13,30,31].

The seventh factor comprised 4 items that assess perceptions of satisfaction with the means/resources and the protocols available in the institution where the respondent works (Table 4), consistent with findings in other studies [12,13,21,22,27].

Finally, the eighth factor of the scale, designated satisfaction with recognition by other professionals, was composed of items referring to the perception of satisfaction with interpersonal relations established with other workers from different professions (Table 4), operating in complementarity [24,25,26,32].

### 3.3. Construct Validity

The correlations between the factors of the Healthcare Professionals’ Job Satisfaction Scale were positive and statistically significant (Table 5). These results indicate that the items belonging to the same factor are homogeneous in content, which confirms the construct validity of the scale.

Although the present study provides evidence of construct validity through exploratory factor analysis and inter-factor correlations, no analysis of convergent or discriminant validity was performed. This decision was due to the exploratory nature of the study, which aimed primarily to develop and identify the underlying dimensional structure of the ESPST. According to psychometric development standards, convergent and discriminant validity assessments are typically conducted during the confirmatory phase, after the factorial model has been established and replicated in independent samples.

Therefore, it is recommended that future research conducts confirmatory factor analysis (CFA) to further assess the convergent and discriminant validity of the ESPST, strengthening its psychometric robustness and applicability in broader healthcare contexts.

### 3.4. Measurement Stability: Intra-Rater Consistency (ICC)

Intra-rater reliability was analysed using the intraclass correlation coefficient (ICC), with the aim of assessing the consistency of measurements made by the same rater at two different points in time. This type of analysis is particularly relevant when it is necessary to ensure that an assessment instrument produces stable and reliable results over time, when applied under the same conditions and by the same examiner.

The results obtained indicated an exceptional degree of reliability. The ICC for single measures was 0.990, with a 95% confidence interval between 0.980 and 0.995, representing an almost perfect level of agreement. The ICC for average measures, which reflects the mean of several measurements, was even higher, at 0.995, with a confidence interval between 0.990 and 0.998 (Table 6).

The associated F-test, F(29, 29) = 204.98; *p* < 0.001, confirmed that variability between participants was significantly greater than residual variability, reinforcing the statistical robustness of the reliability observed (Table 6).

### 3.5. Internal Consistency: Cronbach’s Alpha of the Scale and Subscales

The Cronbach’s alpha coefficient obtained for the total set of items of the ESPST was 0.973, considered excellent.

With respect to the values of Cronbach’s alpha if an item were deleted, these ranged from 0.761 to 0.951. It was decided to retain all items, as their removal would not significantly alter the alpha coefficient of the scale (Table 7).

## 4. Discussion

### 4.1. Development and Validation of the ESPST

The present study aimed to develop and validate the Healthcare Professionals’ Job Satisfaction Scale (ESPST), providing a multidimensional tool for assessing job satisfaction among diverse healthcare categories. The exploratory factor analysis revealed an eight-factor structure explaining 70.31% of total variance, confirming the multidimensional nature of job satisfaction previously reported in the literature [3,11,12,33].

The methodological robustness of the ESPST—demonstrated by the high KMO value (0.963), total variance explained (>70%), and excellent internal consistency (α = 0.973)—aligns with psychometric standards observed in international validation studies [3,34]. Such results confirm the structural soundness of the scale and reinforce its potential use across different cultural and institutional contexts.

The present study aimed to develop and validate the Healthcare Professionals’ Job Satisfaction Scale (ESPST), based on the relevant literature and expert contributions, in order to address the absence of comprehensive instruments capable of assessing, in a multidimensional way, job satisfaction across different professional categories in the healthcare sector.

The exploratory factor analysis revealed a structure composed of eight dimensions, consistent with the theoretical constructs proposed by authors such as Sá [12] and Karaferis et al. [3], who underline the multifactorial nature of job satisfaction.

When compared with international instruments, such as the Job Satisfaction Survey (JSS) developed by Spector (1985) [34] and the Minnesota Satisfaction Questionnaire (MSQ) by Weiss et al. (1967) [35], the ESPST demonstrates conceptual convergence in core domains like leadership, nature of work, and interpersonal relationships. However, it advances beyond these tools by incorporating healthcare-specific dimensions, including satisfaction with institutional protocols, recognition by patients, and interprofessional collaboration—aspects often underrepresented in generic instruments [23,36,37].

Similarly, the Healthcare Employee Satisfaction Scale de Yılmaz & Kevenk (2021) [38] and the Nurses’ Job Satisfaction Scale (ESET) [15] focus primarily on nursing contexts. The ESPST expands their scope, encompassing physicians, operational assistants, and diagnostic and therapeutic technicians, reflecting the complexity of contemporary healthcare teams. This broader applicability strengthens its relevance for interprofessional research and workforce management in healthcare organisations.

Despite the methodological robustness, it is important to recognise that the sample was obtained by convenience and restricted to a single hospital, which conditions the generalisation of the results. Nevertheless, the response rate (31.69% of the target population) and the adequacy of the sample (KMO = 0.963) confer statistical credibility to the study.

The findings corroborate national and international literature, reinforcing the utility of the ESPST as a valid and reliable instrument for assessing job satisfaction in hospital settings. Its regular application may contribute to more precise organisational diagnoses, supporting strategic decisions in the promotion of occupational well-being and quality of care.

The results obtained through exploratory factor analysis revealed a structure composed of eight dimensions, explaining 70.31% of the total variance, a value considered high and indicative of the structural consistency of the scale. These results are in line with previous studies on instruments for assessing job satisfaction in healthcare, which also demonstrate a diversity of factors associated with well-being in the workplace [3,14].

The identification of factors such as “satisfaction with leadership and management”, “satisfaction with colleagues”, and “satisfaction with the nature of work” confirms the relevance of interpersonal relationships, professional recognition, and the alignment between functions and competences for the well-being of healthcare professionals [11].These aspects have been widely recognised in the literature as determinants of motivation, performance, and retention of professionals in healthcare organisations [11,33].

Additionally, the dimension “satisfaction with human resources” is particularly pertinent in a context marked by increasing demands and shortages of professionals in many health services. The perception of adequacy in staffing levels and available resources has also been emphasised as a factor that promotes job satisfaction [37,39].

The strong correlations between the factors confirm the construct validity of the ESPST, supporting the idea that job satisfaction is multidimensional and interdependent. This complexity reinforces the need for comprehensive instruments capable of capturing the different components influencing the work experience of healthcare professionals.

The internal consistency of the scale was high, with a global Cronbach’s alpha value of 0.973, demonstrating excellent reliability of the instrument. All subscales presented values above 0.84, further reinforcing the internal homogeneity of the items and the adequacy of the proposed dimensions. These results are comparable to those reported in other measurement instruments used in similar contexts [14,34].

Despite the promising results, this study presents certain limitations, particularly the convenience sampling and geographical concentration of participants, which may limit the generalisation of the findings. Future research should consider more diverse samples and include confirmatory analyses to consolidate the factor structure identified.

Nevertheless, the ESPST has demonstrated itself to be a valid, reliable, and sensitive instrument for the assessment of healthcare professionals’ job satisfaction in the Portuguese context, and it may constitute a useful tool for research, human resource management, and organisational quality improvement.

### 4.2. Implications for Practice and Health Policy

The validation of the Healthcare Professionals’ Job Satisfaction Scale (ESPST) represents a meaningful advancement for healthcare management and workforce policy, particularly within the Portuguese National Health Service (SNS). As the healthcare sector faces continuous challenges related to staff shortages, ageing professionals, and increasing work demands, having a valid and comprehensive tool to measure job satisfaction is essential for evidence-based decision-making and organisational reform. The ESPST enables institutions to systematically monitor satisfaction levels across different professional categories, supporting the design of tailored interventions that strengthen motivation, retention, and quality of care. These applications are aligned with the Plano Nacional de Saúde 2030 and European directives promoting the sustainability of the healthcare workforce [5,35,36].

At the local policy level, the ESPST can serve as an operational tool for hospital administrators and regional health authorities to identify specific determinants of dissatisfaction—such as leadership issues, lack of recognition, or insufficient human resources—and to formulate targeted human resources strategies. Evidence derived from regular applications of the scale may inform initiatives such as continuous professional development, flexible work arrangements, and recognition systems, which have been shown to improve both satisfaction and service quality [6,9]. Similar approaches have been successfully implemented in other European healthcare contexts, demonstrating that structured monitoring of satisfaction can contribute directly to institutional efficiency and patient outcomes [40].

From a theoretical and global perspective, the ESPST contributes to the international literature by incorporating dimensions that are often neglected in traditional instruments, such as recognition by patients, interprofessional collaboration, and satisfaction with institutional protocols. These elements expand the conceptual framework of job satisfaction in healthcare, integrating both relational and organisational determinants of well-being. Studies by Schneider et al. (2015), Alanazi et al. (2022), and Flindris et al. (2025) have demonstrated the critical role of interprofessional collaboration in predicting job satisfaction, confirming the global relevance of these dimensions for the health workforce [40,41,42].

Moreover, the ESPST’s multidimensional approach allows it to be adapted and validated in different cultural and institutional contexts, contributing to the global harmonisation of job satisfaction assessment. It aligns with international priorities established by the World Health Organization’s Global Strategy on Human Resources for Health 2030 [41] and the Organisation for Economic Co-operation and Development (OECD) framework for resilient health systems [42]. By providing a standardised yet context-sensitive tool, the ESPST can support comparative international research and inform global indicators of workforce well-being, advancing theoretical development in healthcare job satisfaction measurement.

In conclusion, beyond its local applicability, the ESPST serves as both a diagnostic and strategic management instrument, bridging organisational psychology, occupational health, and health policy. Its integration into institutional quality improvement programmes and national monitoring systems could enhance the evidence base for policy interventions, promote equitable work environments, and support the long-term sustainability of healthcare systems.

## 5. Conclusions

This study developed and validated the Healthcare Professionals’ Job Satisfaction Scale (ESPST), providing a psychometrically robust and contextually grounded instrument for assessing job satisfaction among diverse professional categories in healthcare institutions. The results confirmed an eight-factor structure that reflects the multidimensional and interprofessional nature of job satisfaction, encompassing relational, organisational, and professional dimensions.

The findings demonstrate that job satisfaction among healthcare professionals is not limited to individual or occupational factors but emerges from a dynamic interaction between institutional conditions, leadership, teamwork, and recognition—both from peers and patients. By capturing these interconnected elements, the ESPST offers a holistic framework for understanding and measuring professional well-being in healthcare settings.

The methodological rigour and high reliability of the ESPST ensure its applicability across different health institutions, supporting evidence-based human resource management, organisational development, and quality improvement strategies. At the national level, the instrument aligns with the priorities of the Plano Nacional de Saúde 2030, providing decision-makers with an evidence-based tool to identify determinants of job satisfaction, mitigate professional burnout, and promote sustainable workforce policies.

At the international level, the ESPST contributes theoretically to the global discourse on healthcare workforce satisfaction. Its inclusion of dimensions such as interprofessional collaboration, patient recognition, and adherence to institutional protocols positions it as a model for future cross-cultural adaptations and comparative studies. The instrument’s multidimensional approach responds to the World Health Organization’s call for the development of comprehensive tools to support the Global Strategy on Human Resources for Health 2030, thereby strengthening global health system resilience and staff retention.

In summary, the ESPST represents both a scientific and practical contribution: scientifically, it advances the conceptualization of job satisfaction in healthcare by integrating underexplored dimensions; practically, it provides a validated and adaptable instrument capable of informing management decisions and shaping workforce policies. The holistic understanding of job satisfaction proposed in this study reinforces the link between professional well-being, service quality, and patient outcomes—central pillars for building more sustainable and humanised healthcare systems.

## Figures and Tables

**Table 1:** Sociodemographic and professional characteristics of the sample.

Variables	Categories	n	%
Gender	Female	455	82.9
	Male	94	17.1
Age	20–29 years	59	10.7
	30–39 years	167	30.4
	40–49 years	146	26.6
	50–59 years	134	24.4
	60–69 years	43	7.8
Marital status	Single	133	24.2
	Married	260	47.4
	Common-law marriage	87	15.8
	Divorced	58	10.6
	Widowed	11	2.0
Number of children	None	159	29.0
	1–2 children	348	63.4
	>2 children	42	7.7
Academic qualifications	1st Cycle (primary education)	6	1.1
	2nd Cycle	5	0.9
	3rd Cycle	25	4.6
	Secondary education	69	12.6
	Bachelor’s degree	323	58.8
	Master’s degree	118	21.5
	Doctorate	3	0.5
Type of employment contract	Public employment contract	166	30.2
	Permanent individual contract	341	62.1
	Fixed-term individual contract	29	5.3
	Self-employed (“green receipt”)	2	0.4
	Other	11	2.0
Length of professional practice	≤5 years	84	15.3
	6–10 years	88	16.0
	11–15 years	81	14.8
	16–20 years	82	14.9
	21–25 years	58	10.6
	26–30 years	60	10.9
	31–35 years	37	6.7
	36–40 years	48	8.7
	41–45 years	10	1.8
	>45 years	1	0.2
Profession	Nurse	279	50.8
	Operational assistant	106	19.3
	Physician	86	15.7
	Clinical analysis & public health technician	16	2.9
	Cardiorespiratory technician	12	2.2
	Technical Assistant	12	2.2
	Radiology technician	10	1.8
	Physiotherapist	5	0.9
	Occupational therapist	4	0.7
	Pathology technician	3	0.5
	Social worker	3	0.5
	Pharmacist	2	0.4
	Pharmacy technician	2	0.4
	Psychologist	2	0.4
	Speech therapist	1	0.2
	Audiology technician	1	0.2
	Senior diagnostic & therapeutic technician	1	0.2
	Chaplain	1	0.2
Department/Service	Internal medicine	164	29.9
	Support services	88	16.0
	Surgery	74	13.5
	Emergency	73	13.3
	Women’s and children’s health	49	8.9
	Psychiatry & mental health	16	2.9
	Outpatient consultation	13	2.4
	Occupational health & safety	2	0.4
	Several services at the same time	20	3.8
	Other	50	9.1
Function performed	Direct patient care	470	85.6
	Healthcare management	40	7.3
	Administrative tasks	14	2.6
	Laboratory diagnosis	5	0.9
	Information and Public Relations Service	5	0.9
	Auxiliary care	4	0.7
	Care provision & management	3	0.5
	Social services	2	0.4
	Psychosocial support	1	0.2
	Management/leadership	1	0.2
	Spiritual assistance	1	0.2
	Outpatient consultations	1	0.2
	Logistics/courier	1	0.2
Weekly working hours	<35	9	1.8
	35–40	461	91.1
	>40	36	7.1
Work schedule	Shift work	316	57.6
	Fixed schedule	233	42.4
Distance residence–workplace	<30 km	441	80.3
	30–60 Km	81	14.8
	60–100 Km	24	4.4
	>100 Km	3	0.5

**Table 2:** Rotated component matrix of the ESPST.

Factor	Eigenvalue	% Variance	% Cumulative	Rotated Eigenvalue	% Variance	% Cumulative
1	21.760	43.521	43.521	7.709	15.418	15.418
2	3.374	6.747	50.268	5.368	10.735	26.154
3	2.710	5.421	55.689	5.156	10.312	36.466
4	1.945	3.891	59.580	4.200	8.400	44.866
5	1.766	3.532	63.111	4.182	8.364	53.230
6	1.479	2.959	66.070	3.971	7.943	61.172
7	1.105	2.210	68.280	2.681	5.362	66.534
8	1.014	2.029	70.309	1.887	3.775	70.309

**Table 3:** Principal component analysis of the ESPST (Varimax rotation).

Items	1	2	3	4	5	6	7	8	h^2^
35	0.781								0.793
10	0.769								0.693
33	0.763								0.787
14	0.743								0.759
29	0.729								0.750
40	0.704								0.736
5	0.703								0.724
6	0.634								0.596
19	0.572						0.307		0.615
44	0.482		0.424						0.685
23	0.474		0.337				0.444		0.723
21	0.470		0.421						0.648
24	0.401		0.316	0.340					0.574
43	0.342	0.304							0.490
48		0.789							0.823
49	0.320	0.732							0.790
38		0.715		0.329					0.768
50		0.713							0.674
28		0.664							0.707
17	0.316	0.602							0.700
26		0.538		0.302					0.576
9			0.785						0.741
2.			0.772						0.771
4			0.763						0.723
20			0.708						0.699
1			0.697						0.678
18		0.337	0.370	0.319					0.582
16				0.862					0.862
15				0.860					0.864
36				0.790					0.788
34				0.756					0.729
31					0.856				0.836
32					0.844				0.798
45					0.784	0.317			0.784
27	0.342				0.625				0.650
39					0.592				0.458
8	0.428				0.515				0.613
11						0.835			0.842
7						0.806			0.825
46						0.743			0.753
47						0.569			0.645
12						0.485	0.477		0.643
3		0.318	0.314			0.417			0.514
25	0.401						0.632		0.772
30	0.406						0.619		0.715
22						0.388	0.579		0.662
13	0.368	0.334	0.342			0.335	0.373		0.665
41			0.344	0.337				0.590	0.760
37			0.318	0.432				0.572	0.792
42			0.461					0.559	0.786

**Table 4:** Items and factor loadings of the ESPST.

Dimension	Items		Loading
Satisfaction with leadership and management	35	I am satisfied with the encouragement provided by my superiors for professional training.	0.781
	10	I am satisfied with the way in which my superiors give me the opportunity to participate in training/projects.	0.769
	33	I am satisfied with the opportunities for dialogue and information sharing with my superiors.	0.763
	14	I am satisfied that my work is rewarded and/or valued by my superiors.	0.743
	29	I am satisfied with the respect shown by my superiors for the work I do.	0.729
	40	I am satisfied with the suggestions made by my superior to improve my performance.	0.704
	5	I am satisfied with the efforts made by my superiors to improve my working conditions.	0.703
	6	I am satisfied with my participation in decision-making in my workplace.	0.634
	19	I am satisfied with the training opportunities provided by my workplace.	0.572
	44	I am satisfied with the morale and organisational climate in my workplace.	0.482
	23	I am satisfied with the organisation in my workplace.	0.474
	21	I am satisfied with the opportunity to put new knowledge into practice in my workplace.	0.470
	24	I am satisfied with the autonomy I have, within my competences, to provide appropriate care to patients.	0.401
	43	I am satisfied with the protection of my health at work.	0.342
Satisfaction with the nature of work	48	I am satisfied with the work I do on a daily basis.	0.789
	49	I am satisfied to have the opportunity to be in this workplace.	0.732
	38	I am satisfied overall with the work I do.	0.715
	50	I am satisfied with my profession.	0.713
	28	I am satisfied with the duties I perform in my service.	0.664
	17	I am satisfied to perform the role of a healthcare professional in this service.	0.602
	26	I am satisfied with the quality of my work, considering the context in which I perform my duties.	0.538
Satisfaction with colleagues	9	I am satisfied with the trust I can place in my colleagues.	0.785
	2	I am satisfied with the spirit of collaboration between me and my professional peers.	0.772
	4	I am satisfied with the effort shown by my colleagues to provide better care.	0.763
	20	I am satisfied with the competence demonstrated by colleagues in the same profession.	0.708
	1	I am satisfied with the opportunities for dialogue and information sharing with my colleagues.	0.697
	18	I am satisfied with the competences demonstrated by colleagues from professions different from mine.	0.370
Satisfaction with service users’ recognition	16	I am satisfied with the way I am valued by patients and their families.	0.862
	15	I am satisfied that my work is rewarded and/or valued by patients.	0.860
	36	I am satisfied with the respect shown by patients for the work I do.	0.790
	34	I am satisfied with patients’ perception of the work I do.	0.756
Satisfaction with functions and career progression	31	I am satisfied with my salary in relation to the duties I perform.	0.856
	32	I am satisfied with my salary in relation to my skills and knowledge.	0.844
	45	I am satisfied with my salary in relation to the amount of work I do daily.	0.784
	27	I am satisfied with the time I must wait to be promoted in my workplace.	0.625
	39	I am satisfied with the time I must wait until retirement.	0.592
	8	I am satisfied with the opportunities for career progression.	0.515
Satisfaction with human resources	11	I am satisfied with the number of healthcare professionals in relation to the number of tasks to be performed.	0.835
	7	I am satisfied with the number of healthcare professionals in relation to the number of patients to be cared for.	0.806
	46	I am satisfied with the number of colleagues who have the same profession as mine.	0.743
	47	I am satisfied with the number of colleagues in my workplace who have a different profession from mine.	0.569
	12	I am satisfied with the physical conditions of the space where I work.	0.485
	3	I am satisfied with the working hours in my workplace.	0.417
Satisfaction with institutional protocols and resources	25	I am satisfied with the organisation and clarity of the protocols in my service.	0.632
	30	I am satisfied with the number of protocols available to guide service operation.	0.619
	22	I am satisfied with the equipment and materials available in my service.	0.579
	13	I am satisfied with the routines established in the service.	0.373
Satisfaction with recognition by other professionals	41	I am satisfied with the recognition shown by other healthcare professionals for my work.	0.590
	37	I am satisfied with the respect shown by other healthcare professionals for the care I provide.	0.572
	42	I am satisfied with the spirit of collaboration between me and other healthcare professionals.	0.559

**Table 5:** Spearman’s correlations between factor scores of the ESPST.

Variables	(1)	(2)	(3)	(4)	(5)	(6)	(7)
Satisfaction with leadership and management	0.702 **	0.688 **	0.449 **	0.581 **	0.657 **	0.781 **	0.649 **
Satisfaction with the nature of work	1	0.601 **	0.589 **	0.430 **	0.567 **	0.647 **	0.656 **
Satisfaction with colleagues	0.601 **	1	0.428 **	0.409 **	0.476 **	0.627 **	0.685 **
Satisfaction with service users’ recognition	0.589 **	0.428 **	1	0.351 **	0.430 **	0.431 **	0.582 **
Satisfaction with functions and career progression	0.430 **	0.409 **	0.351 **	1	0.557 **	0.510 **	0.408 **
Satisfaction with human resources	0.567 **	0.476 **	0.430 **	0.557 **	1	0.685 **	0.491 **
Satisfaction with institutional protocols and resources	0.647 **	0.627 **	0.431 **	0.510 **	0.685 **	1	0.605 **

Legend: (1) Satisfaction with the nature of work; (2) Satisfaction with colleagues; (3) Satisfaction with service users’ recognition; (4) Satisfaction with functions and career progression; (5) Satisfaction with human resources; (6) Satisfaction with institutional protocols and resources; (7) Satisfaction with recognition by other professionals. Note: ** indicates *p* < 0.01.

**Table 6:** Intra-rater reliability of the ESPST.

	Intraclass Correlation	95% Confidence Interval		F-Test with True Value 0			
		Lower Bound	Upper Bound	Value	df1	df2	Sig
Single measures	0.990	0.980	0.995	204.98	29	29	<0.001
Average measures	0.995	0.990	0.998	204.98	29	29	<0.001

**Table 7:** Internal consistency of ESPST subscales.

Dimension	Items	Item–Total Correlation	Cronbach’s Alpha If Item Deleted
Satisfaction with leadership and management (α = 0.951)	35	0.828	0.946
	10	0.747	0.948
	33	0.835	0.945
	14	0.802	0.946
	29	0.817	0.946
	40	0.767	0.947
	5	0.767	0.947
	6	0.689	0.949
	19	0.693	0.949
	44	0.753	0.948
	23	0.757	0.947
	21	0.720	0.948
	24	0.628	0.950
	43	0.610	0.951
Satisfaction with the nature of work (α = 0.923)	48	0.851	0.902
	49	0.822	0.905
	38	0.817	0.906
	50	0.691	0.919
	28	0.768	0.911
	17	0.748	0.913
	26	0.633	0.923
Satisfaction with colleagues (α = 0.889)	9	0.755	0.861
	2	0.771	0.858
	4	0.725	0.866
	20	0.746	0.863
	1	0.706	0.869
	18	0.531	0.895
Satisfaction with service users’ recognition (α = 0.928)	16	0.849	0.900
	15	0.864	0.895
	36	0.822	0.909
	34	0.790	0.919
Satisfaction with functions and career progression (α = 0.868)	31	0.802	0.824
	32	0.778	0.827
	45	0.754	0.832
	27	0.642	0.850
	39	0.503	0.878
	8	0.576	0.862
Satisfaction with human resources (α = 0.875)	11	0.807	0.832
	7	0.763	0.838
	46	0.761	0.840
	47	0.656	0.858
	12	0.577	0.870
	3	0.523	0.879
Satisfaction with institutional protocols and resources (α = 0.843)	25	0.764	0.761
	30	0.733	0.776
	22	0.592	0.837
	13	0.628	0.822
Satisfaction with recognition by other professionals (α = 0.902)	41	0.830	0.838
	37	0.785	0.876
	42	0.800	0.864

## Data Availability

Due to ethical issues, the data collected and analysed in this study are not available to outside researchers.
